# Genotyping Based on the LTR Region of Small Ruminant Lentiviruses from Naturally Infected Sheep and Goats from Mexico

**DOI:** 10.1155/2019/4279573

**Published:** 2019-05-12

**Authors:** Wolfang P. S. Mendiola, Jorge L. Tórtora, Humberto A. Martínez, María M. García, Sandra Cuevas-Romero, José L. Cerriteño, Hugo Ramírez

**Affiliations:** ^1^Virology, Genetics and Molecular Biology Laboratory, Faculty of Higher Education, Cuautitlan, Veterinary Medicine, Campus 4, National Autonomous University of Mexico, Km 2.5 Carretera Cuautitlán-Teoloyucan San Sebastián Xhala, Cuautitlán Izcalli, MEX, C.P. 54714, Mexico; ^2^Laboratory of Immunovirology, Medical Research in Immunology Unit, Pediatric Hospital, National Medical Center XXI Century, Mexican Institute of Social Security, Mexico; ^3^National Research Center of Animal Microbiology Disciplines, National Research Institute of Forestry and Agriculture, INIFAP, C.P. 05110, Mexico City, Mexico

## Abstract

Small ruminant lentiviruses (SRLVs) belong to the genus* Lentivirus* in the Retroviridae family. There are five genotypes (A, B, C, D, and E), where genotypes A and B have a global distribution and genotypes C, D, and E are limited to Europe. The presence of SRLV has been confirmed in Mexico, with genotype B detected in the central region of the country. We examined the presence of SRLVs and genotype prevalence in 1014 sheep and 1383 goats from 12 Mexican states. Using a commercial competitive ELISA (cELISA) test, we detected SRLV antibodies in 107 sheep (10.55%) and 466 goats (33.69%). We used an endpoint PCR to amplify the LTR region on seropositive animals. A total of 50 sheep and 75 goats tested positive via PCR. Positive amplicons from 11 sheep and 17 goats from ten Mexican States were cloned and sequenced. With the LTR sequence data obtained in this study, a phylogenetic analysis was performed; we also constructed a phylogenetic tree using the obtained sequences and GenBank's available sequences. All studied sequences were associated with genotype B, specifically with the FESC-752 isolate previously identified in Mexico. Highly conserved transcription factor binding sites were observed in analyzed alignments, such as AML (vis), AP-4, and TATA box. However, we identified nucleotide differences at site AP-1 that suggest function loss. Our study found that ovine and caprine genotype B SRLVs are widely distributed in Mexico; a highly conserved LTR region among the sequences evaluated in this study was also found.

## 1. Introduction

Small ruminant lentiviruses (SRLVs) are single-stranded RNA viruses grouped in the order* Ortervirales*,* Retroviridae* family*, Orthoretrovirinae* subfamily, and* Lentivirus *genus [1, 2]. SRLVs have three structural genes (*gag, pol*, and* env*), three accessory genes (*vpr-like*,* vif*, and* rev*) and LTRs at the 5′ and 3′ end of the proviral DNA. The LTR is composed of three regions U3, R, and U5 and have been described as viral transcription promoters, particularly the U3 region that potentializes transcription factor binding sites [3–5]. LTRs can influence viral tropism in the central nervous system when specific CAAAT sequences are duplicated. Poor viral growth occurs when a single CAAAT sequence is present and no transcription occurs when CAAAT is deleted [6]. SRLV infect sheep and goats worldwide, causing multisystemic chronic and progressive infections, characterized by pneumonia, encephalitis, arthritis, and mastitis [7–9]. The first clinical case in sheep, named Maedi-visna virus (MVV), was detected in Iceland in 1930 [9–11]. Cork et al. [12] reported the first* Lentivirus* case in goats in the United States of America and viral isolation was reported in 1980 [13]. Nowadays there are five recognized SRLV genotypes (A, B, C, D, and E), based on nucleotide sequences derived from* gag* and* pol* genes. Initially, genotype A was thought to be exclusive to sheep and genotype B to goats, but interspecies transmission has been well documented [8, 14, 15]. Both genotype A and genotype B (with 15 and 3 subtypes, respectively) are present worldwide. Genotype C was described in Norway, genotype D is found in Switzerland and Spain, and two subtypes of genotype E have been identified in Italian goats [14, 16, 17]. Since the early 1980s, researchers in Mexico have been gathering serological data [18–20], and in 2011 genotype B was first identified in sheep and goats from the state of Mexico [21]. Research on SRLVs became more relevant in Mexico in 2007, following a conflict of economic importance which occurred while exporting sheep to Colombia [22]. In 2016, MVV was recognized as endemic in Mexico, while Caprine Arthritis Encephalitis Virus (CAEV) has been recognized since 1997. To date, there have been no updated studies regarding seroprevalence and genotyping of naturally present SRLVs in sheep and goats throughout Mexico. Thus, which SRLV's LTR genotypes and their genetic characteristics affecting small ruminants populations in Mexico are still unknown. The goal was to identify the prevalence of SRLV genotypes and to study the genetic characteristics of LTRs in infected sheep and goats from different states in Mexico.

## 2. Material and Methods

### 2.1. Animals and Sample Collection

**Figure 1:**
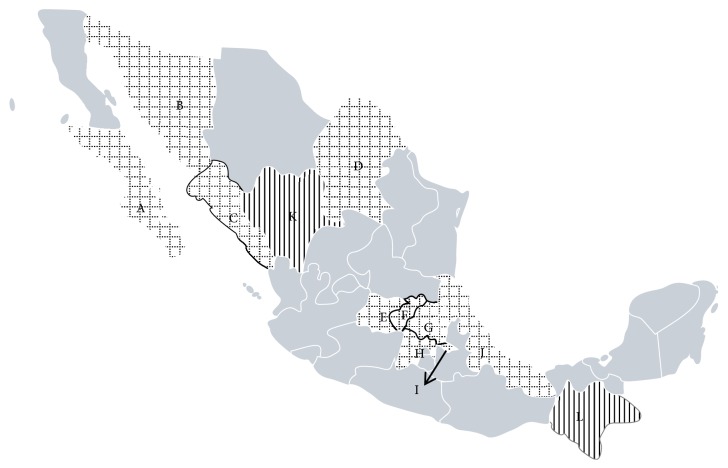
Map of the states in Mexico where samples were gathered. States in vertical lines had no positives results (K, L); states in squares had positive results: K-Durango, L-Chiapas, G-Hidalgo, C-Sinaloa, D-Coahuila, E-Guanajuato, F-Querétaro, I-Tlaxcala, A-Baja California Sur, B-Sonora, H-Estado de México, and J-Veracruz.

We collected blood samples from 1014 sheep (972 females and 42 males) and 1383 goats (1270 females and 113 males) from various states in Mexico (Figure 1). The animals' ages ranged between one and ten years of age. Of the 2397 animals studied, only 43 sheep presented respiratory disease, 14 goats presented arthritis, and 11 goats presented mastitis.

Plasma and peripheral blood leukocytes (PBL) were collected by centrifuging samples at 3500rpm for 15min. Plasma was collected and stored at -70°C for later use, and PBL were processed as described by Gorodezky et al. [23]. DNA from PBL was extracted using a commercial Favorgen® kit (Biotech Corp., Pingtung, Taiwan), quantified by Nanodrop (Lite™, Thermo Scientific™ USA) and stored at -70°C until needed.

### 2.2. Serological Analysis

We tested for SRLVs in plasma using a commercially available cELISA kit (VMRD® USA) detecting gp135 antibodies, following the manufacturer's instructions.

### 2.3. LTR Amplification Using PCR

We used an endpoint PCR to amplify the partial LTR region (U3, U5, and R), using primers designed by Primer3 input v.0.4.0 program. LTRs were detected with a forward 5′-TGTTGCACAGAWTWAGGRACG-3′ and reverse 5′-TCASKGTGACAAAGCAAAATGTAA-3′ primers that amplified a 291bp fragment.

Each sample contained 280 *μ*M dNTPs, 1.5 *μ*M MgCl_2_ (KAPA® Taq Buffer, Roche™, Basel Switzerland), 5 U* Taq* polimerase (Amplificasa®, BIOTECMOL® CDMX, México), 1x buffer, 600 nM of each primer (IDT®, Coralville, IA, USA), and 500 ng of DNA. Amplifications were performed by an initial 95°C/5 min denaturation, followed by 45 cycles (denaturation at 95°C for 30 sec, annealing at 58°C for 30 sec and extension at 72°C for 30 sec) with a final extension at 72°C for 15 min. Final products were separated by horizontal electrophoresis on a 1.5% agarose gel containing 0.5 *μ*g ethidium bromide and visualized using an ultraviolet light transilluminator (UVP® Upland, CA, USA). Amplified samples were gel-purified with a commercial kit (FavorGen®, Bioech Corp., Pingtung, Taiwan) and cloned.

### 2.4. Cloning

To enhance sequencing quality,* E. coli* DH-10*β* competent cells and InsTAclone PCR Cloning kit (Thermo Fisher Scientific™ USA) were used to clone the amplified LTR region. We confirmed molecular cloning using PCR; DNA was extracted from transformed cells using Favorgen® commercial kits (Biotech Corp. Pingtung, Thailand).

Bidirectional sequencing was performed externally at the Biotechnology and Prototype laboratory unit of the FES-Iztacala, UNAM, using the Sanger DNA sequencing method with the ABI 3130x1 (Genetic analyzer, Applied Biosystems®).

### 2.5. Sequence Analysis

A phylogenetic tree was constructed by aligning the obtained nucleotide sequences and comparing them to previously published SRLV genotypes A, B, C, and E sequences (s7631-A4-Suiza-KT453990.1, 697-A3-España-HQ848062.1, P1OLV-A1-Portugal-AF479638.1, kv1772-A1-Islandia-L06906.1, Sa-omvv-A1-Sudafrica-M31646.1, USMARC-A2-USA-KY358787.1, 85/34-A2-USA-AY101611.1, Volterra-B3-Italia-JF502417.1, Fonni-B3-Italia-JF502416.1, FESC-B1-México-HM210570.1, Gansu-B1-China-AY900630.1, Shanxi-B1-China-GU120138.1, OV496-B2-España-FJ195346.1, Caev-Co-B1-USA-M33677.1, 1GA-C-Noruega). Geneious® 11.0.4 program, MrBayes Algorithm, and 1000 bootstrap branches were used for phylogenetic tree construction. Obtained sequences were registered in GenBank with access numbers MK188369 to MK188396.

### 2.6. LTR Region Analysis

LTR region from amplified sequences and reference genotypes A and B sequences were analyzed using the ClustalW program as described by Glaria et al., [7], and transcription factor binding sites and motifs in U3 and R regions were identified.

## 3. Results

### 3.1. Serology and PCR

With the exception of Durango and Chiapas, we detected in all the sampled states a total of 573 seropositive animals, 107 ewes and 466 goats (14 males and 452 females). In goats, 84.8% were younger than 5 years, 4.7% older than 5 years, and 10.5% did not have age information. Thirty-two percent of the sheep were younger than 5 years, but 68% did not have age information. Fifty sheep and 75 goats (1 male and 74 females) were also positive using the PCR test (Table 1). The range of age with more seropositivity results was 2 years; 117 goats and 16 sheep were 2 years old. The positivity rate of the test was 23.9% considering both, goats, and sheep.

### 3.2. Sequences

Twenty-eight nucleotide sequences were obtained, 11 from sheep (one with signs of pneumonia) and 17 from goats (one with arthritis and one with mastitis) from different parts of the country (Figure 1 and Table 1).

### 3.3. Phylogenetic Tree

**Figure 2:**
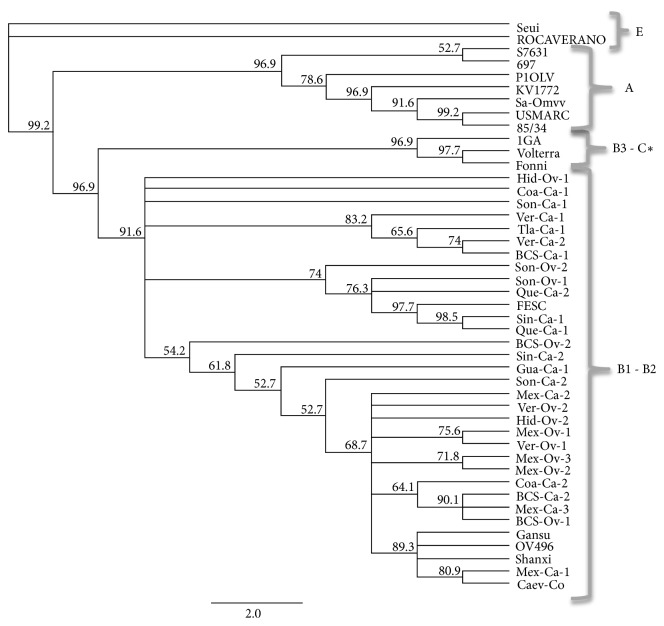
Phylogenetic tree constructed using MrBayes algorithm and the Geneious bioinformatics program. The tree shows significant bootstrap values (higher than 50) that supported the branches. Reference sequences of genotypes A, B, C, and E are named as registered on GenBank® and genotype E is represented as an outgroup. The cloned sequences were named by geographical origin, followed by the species, Ca for goat and Ov for sheep, and finally by the number of the sample per State. Baja California Sur-BCS, Coahuila-Coah, Guanajuato-Gua, Hidalgo-Hid, Estado of México-Mex, Querétaro-Que, Sinaloa-Sin, Sonora-Son, Tlaxcala-Tla, Veracruz-Ver. *∗* Genotypes C and B3 are observed in the same group.

We used the obtained sequences and SRLV reference sequences to construct a phylogenetic tree. We found that all sequences obtained in the study were associated with genotype B. Reference sequences grouped with their corresponding genotype, except for the B3 (Fonni and Volterra genotype sequences), which were grouped with the genotype C sequence (Figure 2).

### 3.4. Analysis of the LTR Region

**Figure 3:**
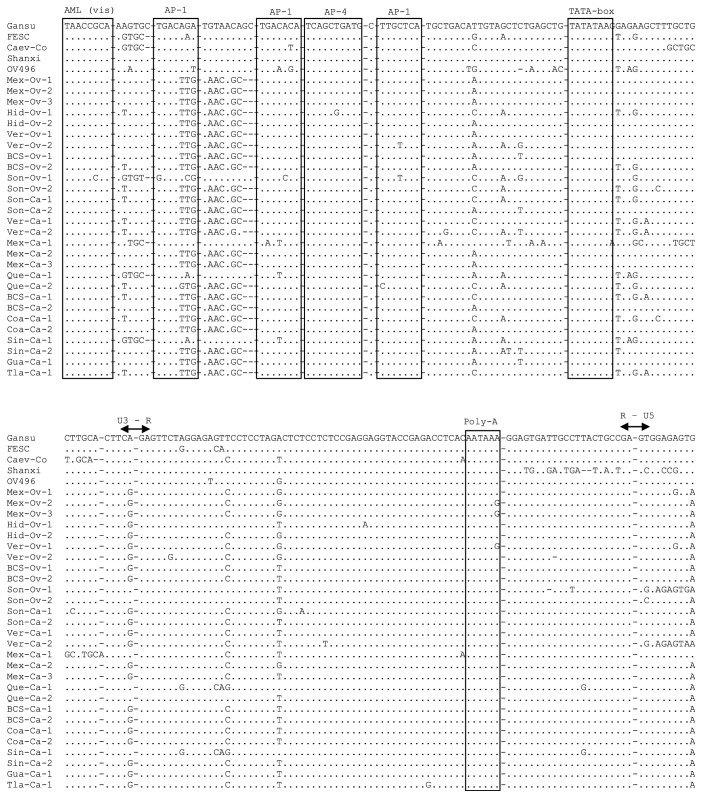
Alignment of genotype B reference sequences and those obtained in this study. Transcription factor binding sites AML (vis), AP-1, and AP-4 are shown in boxes. Minimal changes are observed in the AMLV (vis) and AP-4 sites. A higher number of changes are seen at the AP-1 sites in the sequences. The TATA box is shown in a box; no changes were observed in the sequences. Poly A site is shown in a box showing minimal changes. Limits of the regions U3, R, and U5 are marked.

In the alignment, we observed conserved transcription factor binding sites AP-4, AML (vis), TATA box, and poly A site of the LTR. We also found three AP-1 sites where considerable nucleotide changes were observed, at least in the first copy (Figure 3).

## 4. Discussion

The phylogenetic tree analysis grouped all the Mexican nucleotide sequences together with the reference sequences, and FESC-752 in subtype B. Strain FESC-752 was initially identified in sheep and goats born and bred in the Estado de México and infected with a recombinant strain (CAEV-CO and French strains [21]). Our results demonstrated that SRLV infections are caused by genotype B strains and are widely distributed throughout the country. High genetic similarity to the FESC-752 virus was from 87.1 to 97.2 (data not shown). For genotypes B1 and B2, the range of similarity was from 0.826 to 0.972.

Our findings regarding SRLV genotype B1 infections in goats and sheep may be related to common husbandry methods used in Mexico, particularly the prevalence of mixed herds, which favor the transmission of SRLV in different regions. This is the first description of SRLV genotype B infecting sheep and goats in various states of Mexico.

The analysis of the SRLV LTR region showed two minimal changes in the nucleotide sequences (1 to 2 nucleotides) at the AP-1 sites, and only one copy of AP-4 and minimal changes in a pair of sequences in the AML (vis) site. TATA box and poly A sites were also highly conserved. However, at the first AP-1 site located upstream of the U3 region, changes involving three nucleotides were identified in the majority of the sequences obtained from the study. These could imply a loss of function of this site.

The expression of proviral DNA is favored by transcriptional activation in the LTR, which is carried out and regulated by the binding of transcription factor site to specific sites within the U3 region [3, 24]. These factors are mainly AP-1, AP-4, and AML (vis) sites. By predictive analysis using the TRANSFAC server (Patch1.0 program), the first copy of the AP-1 site could not be located (data not shown) downstream of the sequence in position 19-24 (AGATGT). In our analysis, this site was located in the upstream position 15-21 (TGACAGA); thus all of our study sequences have a complete mutation involving the six nucleotides of this site. This would imply that the site disappears or that it recruits different transcription factors that modify the activity of the region.

LTR analysis was based on work by Glaria et al., [7] in which AP-1 sequences are different from each other (TGACAGA, TGACATA, and TTGCTCA), different sequences have been described in MVV (TGACACA, TAAGTCA TGA [G / C] TCA) and in CAEV (TGAGACA) [24, 25] (Murphy et al. 2006). Sutton et al. [24] stated that the change of bases in the AP-1 sites does not rule out a low affinity to transcription factors and that they can continue to have a basal promoter activity, although alterations in the cis-regulatory function have been observed. All these findings may indicate that the AP-1 site may be functional, even despite sequence changes, since no single consensus sequence exists. AP-1 binding sites have copies other than transcription factor binding sites that would allow transcription regardless of mutations or deletions. By having more copies of these AP-1 sites, the transcript would be multiplied, thus favoring the development of disease [3, 26, 27].

It would be important to perform assays with mutant strains to discern the functionality of AP-1, AP-4, and AML-vis sites found in the Mexican sequences. The predictive analysis also showed that sequence (TGACAG) corresponds to a binding site related to cell differentiation (HOXA9 and Meis-1a and 1b). This finding could be important to better understand the function of region U3 of the LTR and its possible presence in the evolution of the disease.

Juganaru et al. [28] showed that the AP-4 sites are necessary to maintain the basal promoter activity of the LTR in synergy with the AP-1 sites, and that their absence reduces it. The TATA box is also located in the U3 region and it is an important element as a promoter of transcription. TATA box and the AP-4 site are highly conserved among genotypes of SRLV [3, 29], as shown in the sequences obtained in our study. Oskarsson et al. [6] described that more pathogenic strains have more than one AML site (vis) and are very conserved among genotypes. No transcriptional activity is observed when the AML site (vis) is absent [24, 29]. By aligning AML (vis) sites of reference sequences with the nucleotide sequences we obtained in this study, we could identify at least one site. However, it was not possible to determine if there was more than one for complete amplification of the U3 region of LTR was not achieved.

The Mexican SRLV strains were found to be capable of producing clinical pathology. This was verified through sequences obtained from two goats with arthritis, one that also had mastitis, and from a sheep with pneumonia, all from the Estado de México. However, no substantial difference was found in the LTR region of small ruminants with and without clinical signs of disease.

SRLV strains can become slow/low with a decreased capacity for replication due to a lack of transcription factor binding sites or rapid/high strains with an increased replication given by a higher number of transcription factor binding site copies [29]. This was not evaluated* in vitro*, since no viral isolations were made.

Angelopoulou et al. [26, 27] studied the R region for its possible role in viral transactivation and, consequently, the effect it could have on transcription.

These studies have demonstrated the presence of a deletion of 12 to 13 nucleotides in the R region, present in asymptomatic sheep but not in sheep with respiratory signs. This may imply that this region is relevant in the evolution and severity of the disease, particularly in genotype A viruses. No deletions were found in the R region from sequences evaluated in this study and the reference sequences. However, genotype B CAEV-Co sequence was used in both studies as a reference sequence. It would be necessary to perform isolations and replication analyses to reliably demonstrate the replicative capacity of the viruses that were found. It has been suggested that SRLV infections are more common in intensive production systems, with high producing breeds, than they are in nonintensive systems [30].

cELISA tests detected seropositivity in 10.85% of sheep and 33.69% of goats. From these seropositive animals, PCR detected proviral material in 46.72% of sheep and 16.09% of goats. The low number of PCR-positive samples may be due to the high heterogeneity of the SRLV or to low proviral loads that affect test sensitivity [31]. This directly affects the quality and quantity of genetic material PCR amplification, which in turn, impacts the quality of the material to be sequenced. Thus, molecular cloning allows us to ensure genetic material suitable for sequencing. To improve PCR efficiency, a more conserved region between genotypes has been suggested (*pol* and* gag*). These regions could increase the number of positive results in the LTR PCR.

However, PCR is not more sensitive than ELISA tests, which is why the literature suggests the combined use of serological and molecular tests amplifying the breadth of detection of SRLV infection [31].

Finally, one of the first serological studies conducted in Mexico by Adams et al. [18] described seropositive values in less than 10% of goats. Our study established 33.69% of animals were seropositive. Although this study is not an epidemiological study, our results may indicate a trend of increasing prevalence of SRLV infection in goats in Mexico.

## 5. Conclusions

We detected SRLV genotype B in 10 of the 12 sampled states in Mexico, and a broad distribution of the infection throughout the country in both sheep and goats. The LTR region analysis showed highly conserved AP-4, TATA, and AML (vis) sites, all with a single copy. The AP-1 site showed two conserved copies and one mutation copy of three nucleotides were found in most of the Mexican sequences obtained.

## Figures and Tables

**Table 1:** Tested animals, serology, and PCR results, as well as the number of SRLV sequences generated by species and by state.

State	Tested animals		Seropositive animals		PCR positive samples		Sequenced samples	
	Goats	Sheep	Goats	Sheep	Goats	Sheep	Goats	Sheep

BCS	93	69	5	9	2	3	2	2
Chiapas	0	50	0	0	0	0	0	0
Coahuila	114	0	20	0	10	0	2	0
Durango	52	50	0	0	0	0	0	0
Hidalgo	0	182	0	20	0	2	0	2
EM	86	199	48	52	6	33	3	3
Guanajuato	160	0	96	0	11	0	1	0
Querétaro	149	0	117	0	14	0	2	0
Sinaloa	324	71	32	0	6	0	2	0
Sonora	142	181	19	15	2	6	2	2
Tlaxcala	93	160	1	0	1	0	1	0
Veracruz	170	52	128	11	23	6	2	2
Total	1383	1014	466	107	75	50	17	11

BCS= Baja California Sur; EM= Estado de México.

## Data Availability

The sequences used to support the findings of this study have been deposited in the GenBank repository (https://www.ncbi.nlm.nih.gov/nuccore/MK188369- https://www.ncbi.nlm.nih.gov/nuccore/MK188396).
